# A Rare Presentation of Lemmel Syndrome With Pancreas Divisum

**DOI:** 10.1002/ccr3.70210

**Published:** 2025-03-07

**Authors:** Nozha Toumi, Bahaeddine El Ghaieb, Aymen Trigui, Mohammad Saad Saumtally, Kais Fourati

**Affiliations:** ^1^ Department of Radiology, Habib Bourguiba Hospital University of Sfax, Faculty of Medicine Sfax Tunisia; ^2^ Department of General and Digestive Surgery, Habib Bourguiba Hospital University of Sfax, Faculty of Medicine Sfax Tunisia; ^3^ Department of Urology, Habib Bourguiba Hospital University of Sfax, Faculty of Medicine Sfax Tunisia; ^4^ Department of General and Digestive Surgery, Mahrès Regional Hospital University of Sfax, Faculty of Medicine Mahrès Tunisia

**Keywords:** duodenal diverticulum, magnetic resonance cholangiopancreatography, pancreas divisum

## Abstract

Lemmel syndrome, a rare cause of biliary obstruction due to duodenal diverticulum, is even rarer when combined with pancreas divisum.

## 1 Introduction

Lemmel syndrome (LS) is a rare cause of obstructive jaundice due to external compression of the common bile duct (CBD) by a periampullary duodenal diverticulum [1]. Although often asymptomatic, it can sometimes present with abdominal pain or jaundice. Accurate diagnosis requires imaging to rule out common causes like choledocholithiasis or periampullary tumors. The association of LS with pancreas divisum (PD), a congenital defect where the pancreatic ducts fail to fuse, is extremely rare. We report a case of LS coexisting with PD.

## 2 Case Report

A 65‐year‐old woman with no significant past medical history presented with vague abdominal pain persisting for 8 months. She had no fever, jaundice, or other systemic symptoms. Physical examination was unremarkable, with no signs of jaundice.

**FIGURE 1:**
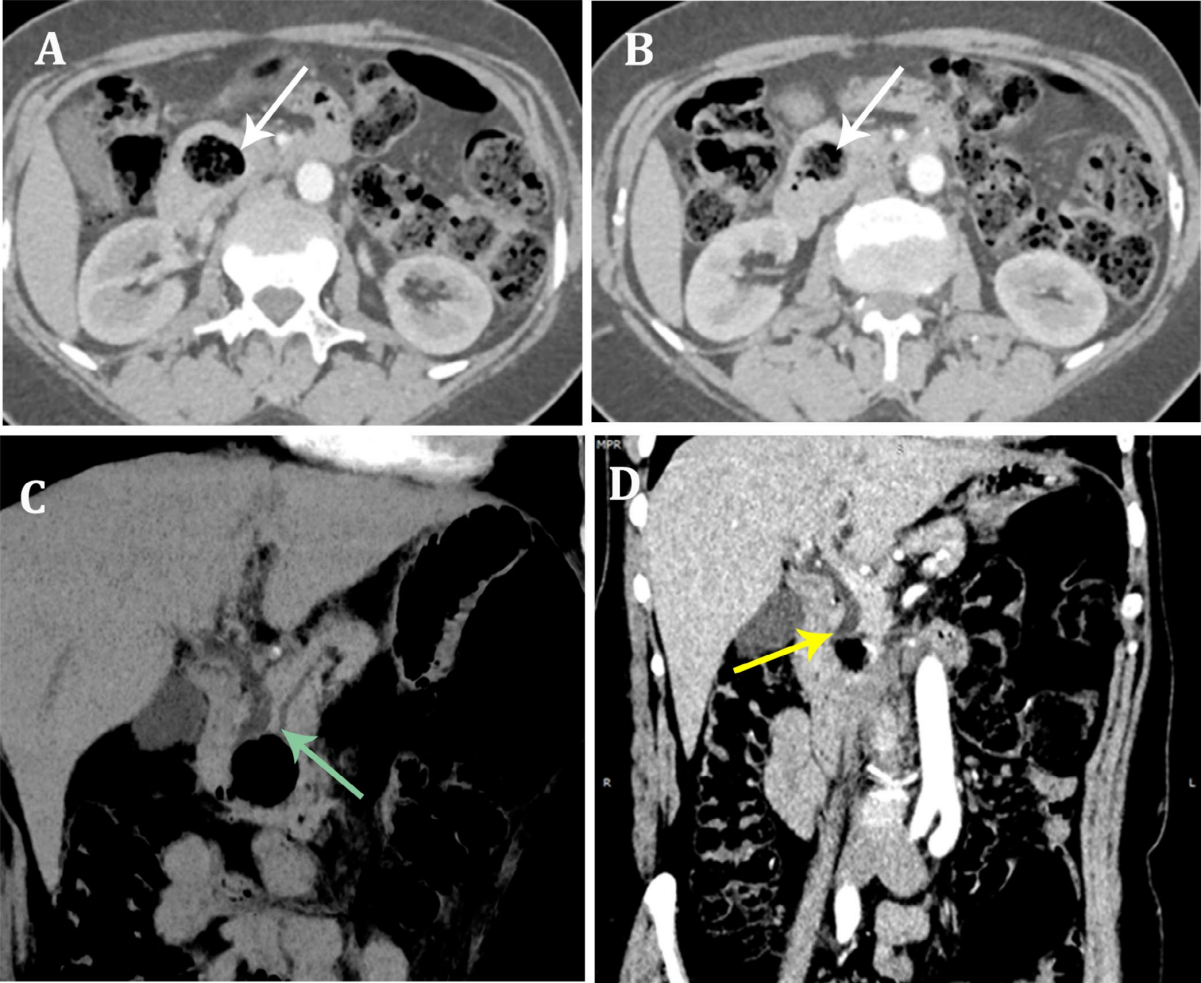
(A and B) Axial CT images showing duodenal diverticulum (white arrows). (C and D) Oblique coronal CT images with non‐dilated main pancreatic duct (green arrow) and external compression of common bile duct by the diverticulum (yellow arrow).

An abdominal CT scan revealed an isolated dilation of the CBD with no dilation of the main pancreatic duct. Additionally, a juxta‐ampullary compressive duodenal diverticulum was identified, exerting a mass effect on the distal CBD. No associated pancreatic duct dilation was noted (Figure 1). Due to these findings, magnetic resonance cholangiopancreatography (MRCP) was performed for further evaluation.

**FIGURE 2:**
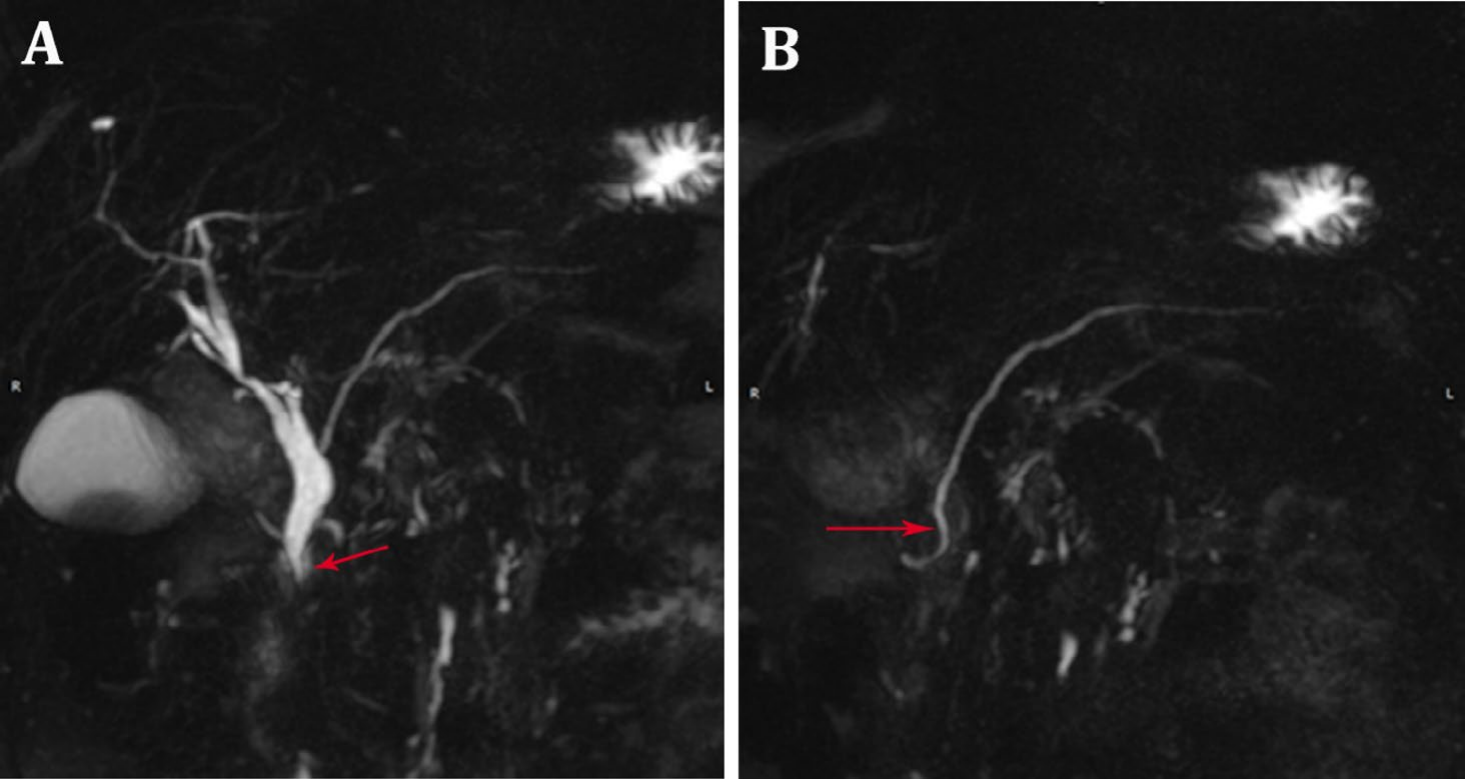
(A) MRCP images showing a dilated common bile duct with pancreas divisum. The small ventral duct joins the distal common bile duct, to open into major papilla along the second part of the duodenum (red arrow). (B) Long dorsal pancreatic duct passing behind the common bile duct opening into minor papilla (red arrow).

MRCP confirmed the presence of the periampullary duodenal diverticulum and its compressive effects on the bile ducts. The diverticulum's contents were hypointense on all imaging sequences. Interestingly, a complete PD was also identified, with the dorsal pancreatic duct draining into the minor papilla and crossing posteriorly to the CBD (Figure 2). No evidence of pancreatic duct dilation or episodes of pancreatitis was found in the patient's history. The lack of associated main pancreatic duct dilation and the separate drainage of the Wirsung duct through the minor papilla likely protected the patient from developing pancreatitis despite the diverticular compression. As the patient was mildly symptomatic, she was successfully treated with analgesics and closely monitored in the outpatient department.

## 3 Discussion

Clinical presentation of LS varies but commonly includes right upper quadrant pain and jaundice, with imaging being crucial in diagnosis. Abdominal ultrasound is often the first imaging modality, typically showing biliary dilation. However, computed tomography (CT) is the gold standard for evaluating duodenal diverticula and their compressive effects. MRCP, as demonstrated in this case, provides a non‐invasive, three‐dimensional evaluation of the biliary and pancreatic systems and helps to exclude other diagnostic possibilities. Management of LS is individualized. Asymptomatic cases may be managed conservatively, potentially with endoscopic sphincterotomy and stenting to alleviate obstruction. Symptomatic cases may require surgical diverticulectomy [2].

The co‐occurrence of LS and PD is rare, with few cases documented [3]. PD results from a failure of the ventral and dorsal pancreatic ducts to fuse during embryonic development. While this anomaly is associated with an increased risk of pancreatitis, no episodes of pancreatitis were observed in this case. The separate drainage of the Wirsung duct through the minor papilla might reduce the mass effect of the diverticulum, potentially explaining the absence of pancreatic complications. In cases of pancreatitis, endoscopic sphincterotomy of the hepatopancreatic ampulla may be considered, and surgical diverticulectomy can be performed to alleviate symptomatic CBD obstruction. However, since the patient did not report debilitating pain or any episode of pancreatitis, a conservative approach was adopted.

## 4 Conclusion

LS and PD are distinct clinical entities, but their association is rare and poses diagnostic challenges. Further awareness of this rare combination can aid clinicians in avoiding unnecessary invasive investigations and optimizing patient management.

## Author Contributions

**Nozha Toumi:** formal analysis, investigation, project administration. **Bahaeddine El Ghaieb:** investigation, writing – original draft. **Aymen Trigui:** conceptualization, writing – original draft. **Mohammad Saad Saumtally:** resources, writing – review and editing. **Kais Fourati:** investigation, writing – review and editing.

## Ethics Statement

The authors have nothing to report.

## Consent

Signed consent from the patient was obtained for publication.

## Conflicts of Interest

The authors declare no conflicts of interest.

## Data Availability

All data underlying the results are available as part of the article and no additional data has been generated.
